# Evaluation of Tele-rheumatology during the COVID-19 Pandemic in Asian Population: A Pilot Study

**DOI:** 10.1155/2021/5558826

**Published:** 2021-09-30

**Authors:** Amit Sandhu, Amit Agarwal, Paramvir Kaur, Meenakshi Sharma, Harnoor Sra, Manvi Singh, Nishant Jaiswal, Anil Chauhan, Anju Gupta, Meenu Singh

**Affiliations:** ^1^Department of Pediatrics, Postgraduate Institute of Medical Education and Research, Chandigarh, India; ^2^Department of Telemedicine, Postgraduate Institute of Medical Education and Research, Chandigarh, India; ^3^Department of Community Medicine and School of Public Health, Postgraduate Institute of Medical Education and Research, Chandigarh, India

## Abstract

**Objective:**

Rheumatoid arthritis (RA) is a chronic autoimmune condition associated with a potential for deformities. It is one of the common conditions to seek health care. Hence, the present study was conducted to assess the telemedicine services for patients suffering from rheumatoid arthritis during the COVID-19 pandemic in an Asian Indian population.

**Methods:**

A prospective study was conducted (March 2020–June 2020) in the telemedicine department of a premier northern Indian tertiary care institution. Out of the total patients enrolled (*N* = 7577) in telemedicine services, 122 rheumatoid arthritis patients (1.6%) were followed for 1 month to assess change in functional status by modified Health Assessment Questionnaire (mHAQ). Telephonic interviews of the enrolled patients were conducted to determine the level of understanding of advice given by consultants, barriers during the consultation, and satisfaction with teleconsultations for rheumatology clinics.

**Results:**

For the native people, language of the clinicians was the main barrier (20%) in telerheumatology. Saving of time and money was observed as beneficial factors for patients. More than three-quarters of all rheumatoid arthritis patients were ready to use teleconsultation in the near future. A similar proportion of patients were in support for the recommendation of these services to other persons.

**Conclusion:**

We report the successful use of telemedicine services in the evaluation and management of rheumatic diseases in the current COVID-19 pandemic situation.

## 1. Introduction

Towards the end of 2019, the world saw the emergence of a new virus that resembled an earlier SARS coronavirus (SARS-CoV). The disease originated in China and within a span of nine months, it had a global presence with more than 210 countries carrying the burden of infection, with India being severely affected. The World Health Organization declared COVID-19 a pandemic, which is a first instance where an infectious disease due to an emerging virus has been declared a pandemic [1]. SARS-CoV-2 is a beta coronavirus with 80% genome resembling SARS-CoV, but it has maximum similarity with horseshoe bat coronavirus [2].

The regular outpatient departments were closed to ensure social distancing to limit the spread of infection. The patients suffering from chronic diseases suffered the most during this period. It has been reported that musculoskeletal disorders, including rheumatoid arthritis (RA), ankylosing spondylitis (AS), osteoarthritis (OA), and psoriatic arthritis (PsA), were the most common reasons to see a health care provider [3]. Although gauging inflammatory joint diseases using teleconsultation is not an ideal approach as these are best diagnosed and analyzed in person, in this pandemic situation, the American College of Rheumatology (ACR) has given the statement that “Tele-health will allow providers to complete assessments and treatments in the patient's residence that would otherwise need transport to the physician office or hospital” [4].

In India, the outdoor patient department (OPD) services were closed in March 2020 [5]. To ease the situation for the patients, teleconsultation services were started in our institute which is a tertiary care, not-for-profit institution in northern India. The present study was conducted to assess the impact of telemedicine services on health-related outcomes in RA patients during lockdown COVID-19 pandemic.

## 2. Methods

### 2.1. Study Subjects

This prospective study was conducted in the Department of Telemedicine of a tertiary care hospital in North India. Details of the patients enrolled via telephone, web portal, application registration, and email from March 2020 to June 2020 were taken. All patients suffering from RA were enrolled and telephonically interviewed by telemedicine staff. The patients, who reported any severe illness related to RA, were contacted by videoconferencing (if needed). During the first telephonic interview, they were also asked about consent for this study, availability of internet and smartphone, and familiarity with WhatsApp and text messaging. These patients were followed after 1 month for assessing their change in functional status and satisfaction from teleconsultation services.

### 2.2. The Questionnaire (Study Tool)

For the functional status, the modified Health Assessment Questionnaire (mHAQ) was used [6, 7]. This is the shortened version of original HAQ with reduced numbers of items than the original. The patients were administered mHAQ in their native language (English/Hindi/Punjabi) telephonically with an instruction to classify all items according to their difficulty level ranging from 0-3. After one month, the same questions were asked from the respective participants, and their answers were recorded.

We developed a questionnaire that focused primarily on evaluating the telerheumatology service's viability and patient's satisfaction levels. Two different reviewers (MS and AG) developed and reviewed the questionnaire for local community reliability and updated it with the recommendations of both healthcare professionals and nonmedical staff. Modified Kuppuswamy scale was used to assess socioeconomic status in urban and rural areas. This scale was created by Kuppuswamy in 1976 and consists of a composite score that includes the education and occupation of the family head along with the income of the family every month, resulting in a score of 3-29 [8]. The level of understanding of advice given by consultant and barriers during consultation was also explored. Treatment expenditure before COVID lockdown, source of information about teleconsultation services, and frequency of OPD visits before COVID lockdown were also recorded. Satisfaction level was assessed on 11-point Likert scale ranging from 0-10. For better understanding of the results, we combine the 0-3, 4-7, and 8-10 points and represent them in the form of not satisfied, somewhat satisfied, and very satisfied, respectively. The questions were asked in their native language (English/Hindi/Punjabi) for the ease of understanding. The data derived from the patient feedback was coded and entered in the MS-Excel sheet for further analysis. The study was approved by institutional ethical committee (NK/6291/Study/021).

### 2.3. Data Analysis

For association in categorical variables, chi-square test of association was used. For nonparametric analysis, the Wilcoxon sign ranked test was used. Statistical analysis was carried out by the “Statistical Package for the Social Sciences” (SPSS Version 20) (IBM SPSS Statistics for Windows, Version 20.0. Armonk; NY), and a *P* value less than 0.05 was considered significant.

## 3. Results

### 3.1. Characteristics of the Participants

**Figure 1:**
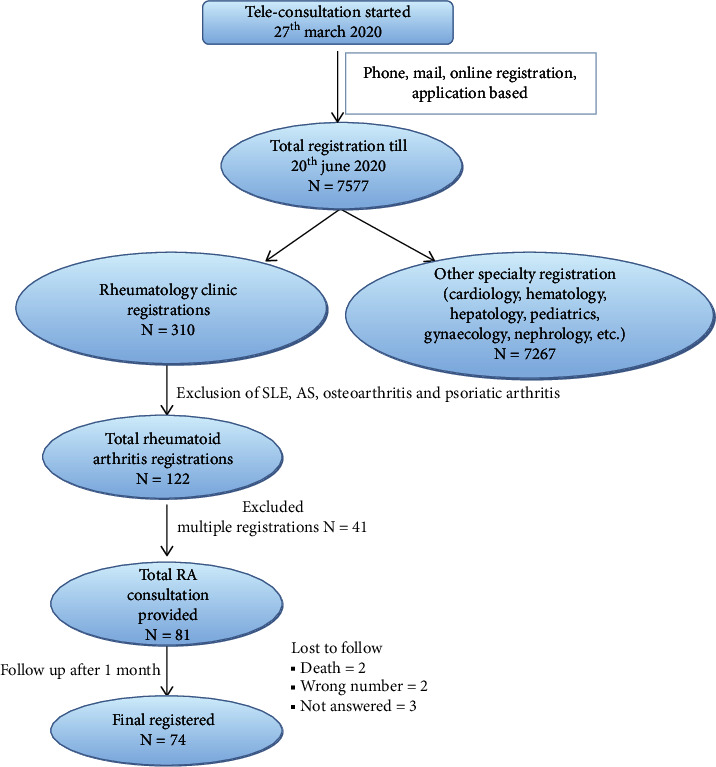
Flow chart of the registrations. Total registrations in the department of telemedicine during lockdown and final registrations in the rheumatology clinic after the exclusion of duplication and lost to follow-up.

A total of 7577 cases were registered for various illnesses from 27^th^ March 2020 to 20^th^ June 2020 in the department of telemedicine (Figure 1). Of these, rheumatological diseases such as rheumatoid arthritis, systemic lupus erythematosus, ankylosing spondylitis, osteoarthritis, and psoriatic arthritis constituted 122, 82, 42, 21, and 23 registrations, respectively. Out of 122 registrations for RA, 41 registrations were excluded due to the multiple registrations. Finally, a total of 81 participants were eligible for enrolment in the study (Figure 1). After one month of follow-up, seven patients were lost to follow-up. In the final 74 participants, 54 (72.9%) were female. Out of 74 patients, 87% (*n* = 65) were earlier enrolled in the OPD. So the diagnosis was already made by the concerned physician. Twelve percent (*n* = 9) patients were new but referred from other nearby hospitals, which were shut due to immediate lockdown at that time. So their diagnosis was made by those referred hospitals.

### 3.2. Functional Status

It was observed that routine activities, such as dressing yourself, including tying shoelaces, doing buttons, getting in/out of the bed, walking on flat ground, wash and dry your body, and getting in/out of the car, showed very significant improvement after one month follow-up (*P* < 0.05). The other mHAQ queries such as lifting a cup, bending down to pick something, and turn faucets on/off also showed improvement after one month but not much significant (Table 1). mHAQ of the previously registered patients (*n* = 65) showed significant improvement in parameters mainly, getting in/out of the bed, bend down to pick up clothing from the floor, and wash and drying of body at three time points (last physical visit, first teleconsultation, and 1-month follow-up) (Supplementary Table 1).

### 3.3. Quality of Life

During lockdown, our hospital broadcast teleconsultation numbers via internet, newspaper, television, and radio. It was observed that *N* = 51 (39%) of the enrolled patients got these helpline numbers via internet followed by newspaper (*N* = 14, 19%) and television (*N* = 1, 1%). Most of the registered patients were dependent on their salaries (*N* = 38, 51%) followed by government reimbursement (*N* = 13, 17%) and health insurances (*N* = 2, 3%). More than half of the patients (*N* = 50, 67%) uses audio services for teleconsultation followed by video (*N* = 15, 20%), application based (*N* = 4, 5%), and text communication (*N* = 5, 6%) (Table 2).

### 3.4. Barriers during Teleconsultation

#### 3.4.1. For the Patients

Language of the clinicians was found to be the main barrier (20%, *N* = 15) during teleconsultation for the patients. This is observed less frequently during direct visits when this hindrance is overcome by the use of physical signs for diagnosis and patient's OPD card for writing prescription. So, at times, it was difficult for the patients to comprehend the medical advice and medicines attributed to unfamiliar technology and illiteracy. To overcome this, WhatsApp and text messages were used. Later patient showed that messaged prescription to pharmacy to avail required medicines. The other barriers noted during teleconsultations were no personal phones (10%, *N* = 8), no internet connection (3%, *N* = 2), and lack of the clarity of advice given by clinicians (23%, *N* = 17) (Table 2).

#### 3.4.2. For the Clinicians

The main difficulties for clinicians include lack of expertise in operating technology and internet connectivity issues in the patient's end which interrupted consultation.

### 3.5. Recommendations and Choice

After all the above mentioned parameters, it was observed that nearly three-fourth of the patients (78.3%) were ready to use teleconsultation in near future. Interestingly, the same proportion of patients (58/74, 78.3%) were in support for the recommendation of these services to other persons (Table 2).

## 4. Discussion

During the current COVID-19 pandemic, it is a great challenge for healthcare workers to follow up and monitor inflammatory diseases like RA, SLE, psoriasis, and juvenile idiopathic arthritis. Telemedicine has possible application in rheumatology, and over previous years, many studies have discussed its applicability and feasibility. In addition, telemedicine is a practical alternative to in-person follow-up rheumatology visits during the covid-19 pandemic [9]. The promising studies in this field are so encouraging that the term “tele-rheumatology” is being used increasingly now [10]. The successful use of a telemedicine initiative by the U.S. Army reveals a possible model for rheumatology treatment in underserved regions [11].

There are several important milestones that were achieved through this study. These include the feasibility of telemedicine in management of rheumatic diseases, and patient's acceptance of telemedicine despite the fact that many of these patients had never seen or interacted over the internet. The acceptance of local physician's willingness to refer patients to the telemedicine clinic was also an achievement to promote telemedicine services.

Previously in a randomized controlled trial of 221 patients of pulmonary, endocrine, and rheumatology clinics, patient satisfaction with physician communication was assessed. They observed that video, teleconsultations (VTC) were noninferior to in-person examination (noninferiority *t*-test *P* = 0.002) [12]. The first national report on rheumatologists in Veterans Affairs (VA) also supports the consideration that rheumatological disorders are suitable for virtual phone visits during the COVID-19 pandemic. This study also reports that preference of telemedicine care and in-person can coexist in an ideal world [9]. In a meta-analysis, the author recorded five feasibility studies, 14 on efficacy, and 9 on satisfaction rates for various synchronous and asynchronous consultations. Moreover, few of these studies found telemedicine to be cost-effective [10].

Time and financial costs are significant barriers in accessing specialist care. Moreover, geographical distribution remains a major barrier to the provision of universal health care [13]. During lockdown, with recession in jobs/salaries, the financial status was highly affected and patients were in much need of a system like teleconsultation which does not increase financial burden on them. In addition, the initial investment in teleconsultation at the end of the organization is related to equipment, workers, need for satellite communications, and dedicated internet which can further increase costs [10].

In the present study, most teleconsultations lasted for 20-25 minutes which included the time taken by the physician to access the patients via the application, screen for signs of disease, documentation, and online consultation via video conference (if needed). There were follow-up calls also, in which most of the participants asked for advices about the change of prescriptions and related complications. These follow-up calls last about 10-15 minutes. This data was supported by previous studies in the US settings in which the teleconsultation eliminates the waiting and travel time drastically from 123 minutes to 20 minutes [14].

High patient satisfaction rates were correlated with telerheumatology visits and recorded as a way to overcome personal expenses and cost of travel time. However, there is evidence that the accuracy of virtual visits was obviously disappointing; with a study showing only 40 percent of correctly diagnosed video teleconference visits, but limited sample size and some methodological issues compromised the reliability of these data [15].

As with all the advancements in technology, there are strengths as well as limitations noted during these consultations. The main limitation was the small sample size; more studies are needed with a bigger sample size and subsequent follow-up. This particular disease, i.e., RA, requires the face-to-face and physical analysis of the patients by the physician, for the determination of disease activity, which is sum of tender joint count and swollen joint count. So the other limitation of telerheumatology is the lack of direct or physical observations. The language and difficulty in understating of the consultation provided by consultants to the patients of rural area are also a weakness of this study, which was resolved to an extent by WhatsApp communication.

## 5. Conclusion

The present study focused on the use of telemedicine services in inflammatory arthritis, specifically rheumatoid arthritis. Our findings conclude that teleconsultation services decrease the time and reduce the financial burden on patients. WhatsApp and text message communications are also necessary along with audio and video consultations in rural or less educated people. However, further elaborated studies are needed to assess the safety, efficacy, and possible incorporation of these services into routine health services beyond the pandemic.

## Figures and Tables

**Table 1:** Modified health assessment questionnaire (MHAQ).

MHAQ	Before (*N* = 74)	After (*N* = 74)	*P* value
Dress yourself, including tying shoelaces and doing buttons	0.5 ± 0.6	0.2 ± 0.5	*P* ≤ 0.001
Get in and out of bed	0.7 ± 0.7	0.4 ± 0.5	*P* ≤ 0.001
Lift a cup of glass to your mouth	0.4 ± 0.7	0.4 ± 0.6	*P* > 0.05
Walk outdoor on flat ground	0.8 ± 0.7	0.6 ± 0.5	*P* ≤ 0.05
Wash and dry your entire body	0.5 ± 0.6	0.3 ± 0.5	*P* ≤ 0.01
Bend down to pick up clothing from the floor	0.7 ± 0.9	0.6 ± 0.8	*P* > 0.05
Turn faucets on and off	0.3 ± 0.5	0.3 ± 0.6	*P* > 0.05
Get in and out of car	0.6 ± 0.7	0.4 ± 0.5	*P* ≤ 0.05

Data is represented as Mean ± SD.

**Table 2:** Demographic characteristics.

Parameters	Level of satisfaction			
	1 (not satisfied)	2 (somewhat satisfied)	3 (very satisfied)	*P* value (*x*^2^, df)
Sex (M : F) *N* = 74	1 : 0	5 : 18	14 : 36	*P* > 0.05 (0.016, 1)
*Occupation scale^#^*				
1 (unemployed)	0	7	18	*P* > 0.05 (0.027, 1)
2 (unskilled)	1	3	7	
4 (skilled)	0	1	6	
5 (clerical)	0	7	13	
6 (semiprofession)	0	4	2	
10 (profession)	0	1	4	
*Per capita family income scale^#^ (in INR)*				
Less than 2390	1	11	22	*P* > 0.05 (1.779, 1)
2391-7101	0	4	19	
7102-11836	0	0	3	
11837-17755	0	5	3	
17756-23673	0	0	2	
23674-47347	0	3	1	
*Source of money spent on treatment (before COVID)*				
Salary	1	9	28	*P* > 0.05 (0.030, 1)
Govt. scheme	0	7	6	
Health insurance	0	0	2	
Others	0	7	14	
*Source of knowledge about teleconsultation services*				
Internet	1	6	22	*P* > 0.05 (1.310, 1)
Newspaper	0	4	10	
TV	0	0	1	
Relatives	0	10	12	
Others	0	3	5	
*Mode of teleconsultation*				
Audio	1	16	33	*P* > 0.05 (0.561, 1)
Video	0	5	10	
App based	0	1	3	
Message	0	1	4	
*Frequency of OPD visit for treatment (before COVID)*				
Weekly	1	3	8	*P* > 0.05 (2.19, 1)
Monthly	0	15	24	
3 monthly	0	3	9	
6 monthly	0	1	1	
Others	0	1	8	
*Choice between OPD and teleconsultation (after COVID)*				
OPD	0	2	14	*P* ≤ 0.05^∗^ (3.63, 1)
Teleconsultation	1	21	36	
*Recommendation of teleconsultation to others*				
Yes	1	18	39	*P* > 0.05 (0.052, 1)
No	0	5	11	
*Barriers during teleconsultation*				
No phone	0	2	6	*P* > 0.05 (1.635, 1)
Language of doctor	0	3	12	
No internet	0	1	1	
Advise not clear	1	4	12	
No problem	0	13	19	

^#^Modified Kuppuswamy scale was used for calculation of occupation and per capita family income; source of money, source of knowledge about teleconsultation, and frequency of visit in OPD were noted during the baseline teleconsultation; choice between OPD/tele, recommendation to others, and barriers were determined after follow-up during feedback call; mode was checked during their first and follow-up consultation. ^∗^*P* value less than 0.05 is considered significant.

## Data Availability

All the required data is already provided in manuscript. Other raw data can be provided on special request.

## Supplementary Materials

Supplementary Materials Table 1: statistical analysis of mHAQ in previously registered patients. Data is represented as Mean ± SD.
