# Supplementary material for: Evaluation of Tele-rheumatology during the COVID-19 Pandemic in Asian Population: A Pilot Study
Source: Int J Telemed Appl. 2021 Sep 30;2021:5558826. doi: 10.1155/2021/5558826 (PMC8497142; doi:10.1155/2021/5558826)
Supplement: Supplementary Materials — Table 1: statistical analysis of mHAQ in previously registered patients. Data is represented as Mean ± SD. [file 5558826.f1.docx]

| **MHAQ** | **Last Physical Visit**  **(N=65)** | **Baseline**  **(N=65)** | **Follow up 1**  **(N=65)** | **P Value** |
| --- | --- | --- | --- | --- |
| **Dress Yourself, including tying shoelaces and doing buttons** | 0.6 ± 0.6 | 0.6 ± 0.6 | 0.4 ± 0.6 | P > 0.05 |
| **Get in and out of bed** | 0.9 ± 0.8 | 0.7 ± 0.8 | 0.5 ± 0.6 | P < 0.05 |
| **Lift a cup of glass to your mouth** | 0.5 ± 0.8 | 0.4 ± 0.7 | 0.7 ± 0.8 | P ≤ 0.05 |
| **Walk outdoor on flat ground** | 0.8 ± 0.8 | 0.8 ± 0.8 | 0.7 ± 0.6 | P > 0.05 |
| **Wash and dry your entire body** | 0.7 ± 0.6 | 0.6 ± 0.6 | 0.4 ± 0.6 | P < 0.05 |
| **Bend Down to pick up clothing from the floor** | 0.7 ± 0.9 | 0.7 ± 0.9 | 0.5 ± 0.7 | P < 0.05 |
| **Turn faucets on and off** | 0.3 ± 0.6 | 0.3 ± 0.6 | 0.6 ± 0.8 | P < 0.05 |
| **Get in and out of car** | 0.6 ± 0.7 | 0.5 ± 0.7 | 0.4 ± 0.5 | P < 0.05 |

**Table 1:** Statistical analysis of mHAQ in previously registered patients

Legend: Data is represented as Mean ± SD
